# Integrating uterine microbiome and metabolome to advance the understanding of the uterine environment in dairy cows with metritis

**DOI:** 10.1186/s42523-024-00314-7

**Published:** 2024-05-27

**Authors:** S. Casaro, J. G. Prim, T. D. Gonzalez, F. Cunha, R. S. Bisinotto, R. C. Chebel, J. E. P. Santos, C. D. Nelson, S. J. Jeon, R. C. Bicalho, J. P. Driver, Klibs N. Galvão

**Affiliations:** 1 Department of Large Animal Clinical Sciences, University of Florida, Gainesville, FL USA; 2 Department of Clinical Sciences, Auburn University, Auburn, AL USA; 3 Department of Animal Sciences, University of Florida, Gainesville, FL USA; 4 D. H. Barron Reproductive and Perinatal Biology Research Program, University of Florida, Gainesville, FL USA; 5 Department of Veterinary Biomedical Sciences, Long Island University, Brookville, NY USA; 6 FERA Diagnostics and Biologicals, College Station, TX USA; 7 Division of Animals Sciences, University of Missouri, Columbia, MO USA

**Keywords:** Microbiome, Metabolome, Multi-omics, Metritis, Uterine disease, Dairy cows

## Abstract

**Background:**

Metritis is a prevalent uterine disease that affects the welfare, fertility, and survival of dairy cows. The uterine microbiome from cows that develop metritis and those that remain healthy do not differ from calving until 2 days postpartum, after which there is a dysbiosis of the uterine microbiome characterized by a shift towards opportunistic pathogens such as Fusobacteriota and Bacteroidota. Whether these opportunistic pathogens proliferate and overtake the uterine commensals could be determined by the type of substrates present in the uterus. The objective of this study was to integrate uterine microbiome and metabolome data to advance the understanding of the uterine environment in dairy cows that develop metritis. Holstein cows (*n* = 104) had uterine fluid collected at calving and at the day of metritis diagnosis. Cows with metritis (*n* = 52) were paired with cows without metritis (*n* = 52) based on days after calving. First, the uterine microbiome and metabolome were evaluated individually, and then integrated using network analyses.

**Results:**

The uterine microbiome did not differ at calving but differed on the day of metritis diagnosis between cows with and without metritis. The uterine metabolome differed both at calving and on the day of metritis diagnosis between cows that did and did not develop metritis. Omics integration was performed between 6 significant bacteria genera and 153 significant metabolites on the day of metritis diagnosis. Integration was not performed at calving because there were no significant differences in the uterine microbiome. A total of 3 bacteria genera (i.e. *Fusobacterium, Porphyromonas*, and *Bacteroides*) were strongly correlated with 49 metabolites on the day of metritis diagnosis. Seven of the significant metabolites at calving were among the 49 metabolites strongly correlated with opportunistic pathogenic bacteria on the day of metritis diagnosis. The main metabolites have been associated with attenuation of biofilm formation by commensal bacteria, opportunistic pathogenic bacteria overgrowth, tissue damage and inflammation, immune evasion, and immune dysregulation.

**Conclusions:**

The data integration presented herein helps advance the understanding of the uterine environment in dairy cows with metritis. The identified metabolites may provide a competitive advantage to the main uterine pathogens *Fusobacterium, Porphyromonas and Bacteroides*, and may be promising targets for future interventions aiming to reduce opportunistic pathogenic bacteria growth in the uterus.

**Supplementary Information:**

The online version contains supplementary material available at 10.1186/s42523-024-00314-7.

## Background

Metritis affects around 25% of Holstein cows shortly after calving [1], impacting production, reproduction, culling, and welfare [2–4]. Metritis is characterized by a dysbiosis of the uterine microbiome in which opportunistic pathogenic bacteria such as *Fusobacterium*, *Porphyromonas*, and *Bacteroides* overtake the uterine commensals such as *Mycoplasma* and *Ruminococcus* [5–7]. Opportunistic pathogens are characterized as organisms that can become pathogenic following a perturbation to their host. Interestingly, the uterine microbiome from cows that develop metritis and those that remain healthy do not differ from calving until 2 days postpartum [6]. Therefore, whether opportunistic pathogenic bacteria proliferate and overtake the uterine commensals could be determined either by how efficiently the immune system respond to invading pathogens [8, 9] or by the type of substrates present inside the uterus shortly after calving. For instance, lesser abundance of lactic acid has been identified in the uterus of cows with metritis when compared with cows without metritis [10]. Lactic acid is one of the main substrates for *Fusobacterium necrophorum* growth [11]. Lactic acid utilizing bacteria such as *Fusobacterium necrophorum* can also metabolize the amino acid tryptophan into indole and its derivatives, which can serve as bacterial signaling molecules, effectively regulating virulence, biofilm formation, motility, and sporulation, thereby inhibiting the growth of commensal bacteria [12, 13]. The indole derivative, indole-3-acetate has been shown to be greater in the uterus from cows that develop metritis when compared with cows without metritis [10]. Therefore, it is possible that the uterine metabolome may promote the growth of opportunistic pathogenic bacteria, which consequently may inhibit the growth of uterine commensals leading to uterine dysbiosis and metritis development.

Although previous research focused on the difference in uterine microbiome [5, 6] or metabolome [10] between cows that developed metritis and cows that did not, little is known about the microbiome-metabolome interactions in the uterus of cows that develop metritis. Therefore, the hypothesis of the current study was that specific uterine metabolites are associated with specific bacteria genera involved in the development of metritis. Hence, the objectives of the study were to compare the uterine microbiome and metabolome at calving and on the day of metritis diagnosis between cows that developed metritis and cows that did not and integrate both omics datasets to advance the understanding of the uterine environment in dairy cows with metritis.

## Methods

This case-control observational study was conducted at the University of Florida Dairy Unit from September 2019 to March 2020.

The cows used for this study were a subset of cows used in a previous study [9]. Analyses of uterine microbiome [6] and serum [10, 14] metabolome using principal component analysis, principal coordinate analysis (PCoA), and partial least square discriminant analysis (PLS-DA) encompassing 12 to 24 cows per group were able to depict statistical differences; therefore, the inclusion of a larger number of cows per group (*n* = 52) was expected to ensure sufficient power for the characterization of changes in the uterine microbiome and metabolome associated with metritis in the current study.

### Cows, Housing, and feeding

A total of 128 Holstein cows, consisting of 71 primigravid (i.e. pregnant nulliparous) and 57 multigravid (i.e. pregnant primiparous or multiparous) cows, were included in the study which started at 260 days of gestation and ended at 13 ± 1 days after calving. Throughout the study period, primigravid and multigravid cows were housed in separate naturally ventilated barns with sand-bedded free-stalls. In the prepartum phase, primigravid and multigravid cows were provided with a total mixed ration (TMR) twice daily formulated to either meet or exceed their nutritional requirements [15]. On a dry basis, the TMR for primigravid was composed of 93.1% organic matter, 15.8% crude protein, 34.8% neutral detergent fiber, 41.0% non-fiber carbohydrates, 4.40% ether extract, 0.62% calcium, 0.28% phosphorus, 0.32% magnesium, 1.19% potassium, 0.24% sulfur, 0.13% sodium, and 0.97% chloride. On a dry basis, the TMR for multigravid was composed of 92.3% organic matter, 14.7% crude protein, 43.7% neutral detergent fiber, 31.1% non-fiber carbohydrates, 2.8% ether extract, 0.64% calcium, 0.33% phosphorus, 0.47% magnesium, 1.49% potassium, 0.40% sulfur, 0.11% sodium, and 0.86% chloride.

After calving, primiparous and multiparous cows were fed a TMR formulated to meet or exceed their nutrient requirements for lactating Holstein cows producing 45 kg of 3.5% fat-corrected milk [15] twice daily. On a dry basis, the postpartum TMR for primiparous was composed of 92.4% organic matter, 18.4% crude protein, 23.5% neutral detergent fiber, 45.7% non-fiber carbohydrates, 5.4% ether extract, 0.64% calcium, 0.39% phosphorus, 0.33% magnesium, 1.60% potassium, 0.16% sulfur, 0.41% sodium, and 0.39% chloride. On a dry basis, the postpartum TMR for multiparous was composed of 91.0% organic matter, 17.4% crude protein, 31.6% neutral detergent fiber, 37.3% non-fiber carbohydrates, 4.0% ether extract, 1.00% calcium, 0.38% phosphorus, 0.40% magnesium, 1.65% potassium, 0.21% sulfur, 0.55% sodium, and 0.66% chloride. Throughout the postpartum period, all cows were milked twice daily at 0600 and 1800 h. Meteorological data was obtained from The Weather Underground, Inc. (https://www.wunderground.com/) for the city of Hague, Florida, and was used to calculate the temperature-humidity index. The average monthly temperature-humidity index from September 2019 to March 2020 were 76.2, 72.4, 59.3, 59.8, 57.9, 58.5, and 66.1, respectively. The yearly rolling herd average milk yield was approximately 11,000 kg during the course of the study.

### Calving related information

At calving, all cows were scored for body condition (BCS) using a scale from 1 to 5 [16] and body weight (BW). The BCS at calving for cows that did and did not develop metritis was 3.48 ± 0.39 and 3.59 ± 0.38, respectively. The BW at calving for cows that did and did not develop metritis was 664 ± 81 kg and 681 ± 82 kg, respectively. Vulvovaginal laceration was scored (VLS) as previously described [17]: 0 = no laceration; 1 = laceration less than 2 cm at the dorsal commissure of the vulva or the lateral walls of the vulva/vagina, or both; 2 = laceration greater than 2 cm at the dorsal commissure of the vulva or the lateral walls of the vulva/vagina, or both. Cows with a VLS score of 2 were classified as having a calving-related disorder. Information about calving ease, stillbirth, and twins were recorded. Calving ease was defined as 0 = unassisted calving; 1 = slight assistance; 2 = dystocia. Stillbirth was defined as the birth of a dead calf or a calf that died within 24 h of birth. Cows were evaluated the day after calving to determine and record the occurrence of retained fetal membranes, characterized by failure to release the placenta within 24 h of calving. Cows that had dystocia, stillbirth, vulvovaginal laceration, retained fetal membranes, and/or twins were considered to have had a calving related disorder (CRD). A total of 53 cows had a CRD, of which 32 developed, and 21 did not develop metritis.

### Exclusion criteria

Cows that developed mastitis, digestive problems, or respiratory disease in the first 35 days after calving, cows that received antimicrobial treatment before metritis diagnosis, and cows diagnosed with metritis after 10 days after calving were excluded from the study. Cows that developed metritis after day 10 were excluded because sampling was only performed up to day 10. Exclusion of cows diagnosed with metritis after day 10 ascertained that these cows would not be misclassified as healthy cows in our analysis. A total of 13 cows were excluded. Four cows were excluded because they were treated with antimicrobials before metritis diagnosis. Three cows were excluded because of death. One cow was excluded because of uterine torsion and one cow was excluded because of peritonitis. Four cows were excluded because they were diagnosed with metritis at 13 ± 1 days after calving. After exclusions, there were 115 cows available; 52 that did and 63 that did not develop metritis.

### Uterine discharge evaluation

Uterine discharge was evaluated using a Metricheck device (Metricheck, Simcro, New Zealand) at 3 ± 1, 7 ± 1, 10 ± 1 and 13 ± 1 days after calving using a 5-point scale as previously described [5]: 1 = not fetid normal lochia, viscous, clear, red, or brown; 2 = cloudy, pink, red, or brown mucoid discharge with flecks of pus; 3 = not fetid, pink red or brown mucopurulent discharge with < 50% pus; 4 = not fetid, pink, red or brown purulent discharge with ≥ 50% pus; 5 = fetid red-brownish, watery discharge.

### Cases definition and control selection

Cows with a uterine discharge score of 5 in at least one examination were classified as having metritis and cows with a discharge score ≤ 4 were classified as not having metritis. Cows diagnosed with metritis up to day 10 were considered cases for this study. There were 52 metritis cases, and 10, 23, and 19 cows were diagnosed with metritis on day 3, 7, and 10, respectively. Control cows (*n* = 52) that did not develop metritis were matched with 52 cows that developed metritis, hence, 104 cows were used for bioinformatics and statistical analyses. An attempt was made to pair cows that did and did not develop metritis according to the day of metritis diagnosis, although it was not always possible. Of the control cows, 10, 32, and 10 cows were sampled on day 3, 7, and 10, respectively. Of these 52 cows, 14, 18, 13, and 7 cows had a uterine discharge score of 1, 2, 3, and 4, respectively.

### Uterine fluid collection

All cows had uterine fluid collected at calving (first 24 h after calving), and at diagnosis of metritis. Briefly, the cow’s cervix was stabilized by rectal palpation, the vulva was rinsed with alcohol 70% (vol/vol) and dried with paper towels. Subsequently, a single-use plastic round-tip pipette (UterFlush pipettes, Van Beek) was introduced into the vagina at a 45° angle and manipulated through the cervix. A total of 50 mL of sterile saline solution (0.9% sodium chloride irrigation, Baxter) was infused into the uterine lumen using a 60-mL syringe (Covidien) attached to the end of the pipette. Uterine contents were homogenized, retrieved into the same 60-mL syringe, and transferred to a sterile 15-mL conical tube (VWR). After collection, tubes were placed on ice and transported to the laboratory within 2 h. Once in the laboratory, uterine fluid samples were aliquoted into 2-mL microcentrifuge tubes (Eppendorf) and stored at -80 ^o^C until essayed.

#### Microbiome analysis

One frozen uterine fluid aliquot was submitted to FERA Diagnostics and Biologicals Corporate in College Station, Texas for microbiome analysis. Samples were analyzed by technicians blinded to study groups. DNA extraction was performed using a Mag-Bind Universal Pathogen 96 Kit (Omega Bio-Tek, Norcross, GA) in accordance with manufacturer instructions. The 16 S rRNA gene was amplified by PCR. Amplification of the V4 hypervariable region of the bacterial/archaeal 16 S rRNA gene was performed as previously described [18] using the Illumina MiSeq platform (Illumina Inc.). Description of PCR and thermocycler conditions are available in https://earthmicrobiome.org/protocols-and-standards/16s/. After DNA amplification, electrophoresis using 1.2% (wt/vol) agarose gels stained with 0.5 mg/mL ethidium bromide was used to verify amplicon presence and size. DNA purification was carried out using magnetic beads Mag-Bind TotalPure NGS (Omega Bio-Tek, Norcross, GA) in accordance with manufacturer instructions. Samples were standardized to the same concentration and pooled into a run for library preparation and sequencing, which was performed using the MiSeq Reagent Kit v2 (300 cycles) on the MiSeq platform (Illumina Inc.).

Non-biological nucleotides were removed, and raw sequenced amplicons were analyzed using the DADA2 package of RStudio Version 2023.06.1 + 524 (RStudio, PBC, Boston, MA) following the DADA2 Pipeline Tutorial (https://benjjneb.github.io/dada2/tutorial.html). After filtering and trimming, the amplicon sequence variant (ASV) table was constructed. Then, chimeric reads were removed, and the number of reads were standardized to the median read number of all the samples. Taxonomy was assigned to ASV using the Greengenes database (http://greengenes.lbl.gov).

#### Total Bacteria 16 S rRNA gene quantification

The total bacterial 16 S rRNA gene quantification was carried out using the Femto™ Bacterial Quantification Kit (Zymo Research Corp, Irvine, CA) according to the manufacturer’s instructions. First, DNA extracts were diluted to 1:10 prior to quantification. Briefly, 18 µL of the kit’s master mix was added to each well with 2 µL of each sample. The PCR cycling condition consisted of 95 °C for 10 min for initial denaturation, 40 cycles of 95 °C for 30 s (denaturation), 50 °C for 30 s (annealing), and 72 °C for 1 min (extension), followed by a final extension of 72 °C for 7 min. The amount of DNA in each sample was calculated based on the standard curve. Data for total 16 S rRNA are described as nanograms of 16 S rRNA per mL. All samples were run in duplicate. Intra-assay coefficient of variation for plates 1 to 8 were 1.01, 0.35, 1.34, 0.45, 2.80, 0.40, 0.28, and 0.61%, respectively. The inter-assay coefficient of variation was 0.91%. While relative abundance provides insights into the proportional representation of bacterial species within a sample, estimated absolute counts offer information on the actual quantity or abundance of individual bacteria. Therefore, estimated bacterial counts were calculated multiplying the total bacterial 16 S rRNA by the relative abundance of each bacterial genus. Logarithms to the base 10 conversions of the raw values were then determined.

### Metabolome Analysis

The second frozen uterine fluid aliquot was submitted to the University of California West Coast Metabolomics Center in Davis, CA for metabolome analysis. Samples were analyzed by technicians blinded to study groups using untargeted gas chromatography with time-of-flight mass spectrometry in a single batch as previously described [19, 20]. The carrier selected was helium gas, and a column comprised of 95% dimethyl/5diphenyl polysiloxanesne was used. The column flow rate was set at 1 mL/minute, and the initial oven temperature was set at 50 °C followed by a 20 °C increase per min up to a final temperature of 330 °C, which was held constant for a period of 5 min. Injection temperature was set to begin at 50 °C followed by a 12 °C increase per second up to 250 °C. Retention of primary metabolites was evaluated using default settings from ChromaTOF v. 2.32 and quantification was reported as peak height. Each metabolite was identified based on its mass and charge relationship. Metabolites were annotated using PubChem, Kyoto Encyclopedia of Genes and Genomes, and Human Metabolome Database. Of the 873 detected metabolites, a total of 253 metabolites were annotated, and 620 were unknown (Supplemental Table S1).

### Statistical analyses

Differences in metabolites and bacteria genera associated with metritis were analyzed at each timepoint separately using RStudio Version 2023.06.1 + 524 (RStudio, PBC, Boston, MA).

For microbiome analysis, alpha diversity was evaluated calculating Shannon and Simpson indexes using the estimate_richness function of the phyloseq package. The association between metritis and each index was analyzed using the Wilcoxon test from the stats package. To assess beta diversity, permutational analyses of variance (PERMANOVA) were performed based on Bray-Curtis distances with 9,999 permutations using the Adonis2 function of the vegan package. The full models included the effects of metritis (metritis vs. no metritis), parity (primiparous vs. multiparous), CRD (yes, no), and their interactions. To account for day of metritis diagnosis and matching of control cows, the model also included the effect of day (d3, d7, d10). A backward elimination procedure was applied when CRD, an interaction containing CRD, or day of diagnosis had a *P* > 0.05. To visualize the differences in bacteria genera associated with metritis, PCoA with Bray-Curtis distances were performed using the ordinate function of the phyloseq package. The significance and effect size of individual bacteria genera were investigated as both relative abundance and estimated counts by performing Wilcoxon tests with Bonferroni corrections followed by Linear discriminant analysis Effect Size (LEfSe) using the lefser function of the lefser package. To assess the association between vaginal discharge scores (1 to 5) and the main individual bacteria genera estimated counts and relative abundance, Wilcoxon tests from the stats package with Bonferroni corrections were performed.

For metabolome analysis, metabolites were first log-transformed and auto-scaled. To analyze differences in the plasma metabolome between metritis and parity on each timepoint, PERMANOVA were performed based on Euclidean distances with 9,999 permutations using the Adonis2 function of the vegan package. The models included the effects of metritis (metritis vs. no metritis), parity (primiparous vs. multiparous), CRD (yes, no), and their interaction. To account for day of metritis diagnosis and matching of control cows, the model also included the effect of day (d3, d7, d10). A backward elimination procedure was applied when CRD, an interaction containing CRD, or day of diagnosis had a *P* > 0.05. To visualize the differences in metabolites associated with metritis, PLS-DA were performed using the splsda function of the mixOmics package. When an effect of metritis was observed (PERMANOVA *P* ≤ 0.05), the significance and effect size of individual metabolites were investigated by performing Wilcoxon tests with Bonferroni corrections followed by LEfSe using the lefser function of the lefser package.

Microbiome and metabolome data were then integrated (i.e. analyzed together) to explore how microbial counts and metabolites relate to each other, providing a more comprehensive understanding of their interactions within the uterine environment. Microbiome and metabolome data integration was performed between estimated microbial counts and metabolites with a *P* ≤ 0.05 to the Bonferroni corrected Wilcoxon tests using the Data Integration Analysis and Biomarker discovery using Latent variable approaches for Omics studies (DIABLO) function of the mixOmics package [21]. Briefly, DIABLO is a multivariate integrative classification method created to identify correlated or co-expressed variables from heterogeneous datasets. Herein, the N-integration supervised Sparse PLS-DA approach for variable selection was performed to identify latent structures composed of strongly correlated variables across both datasets [22, 23]. Firstly, optimal number of components and variables were selected with the perf function of the mixOmics package using cross-validation with 10 folds and 100 repetitions. Then, the block.splsda function was performed on the selected variables to assess the correlation between the bacteria genera and metabolites able to differentiate among cows with and without metritis as previously described [24]. Lastly, the network function from the mixOmics package was used to export the correlation networks, which were then edited for presentation purposes using Metscape 2 [25] within the CytoScape 3.8 platform. Because there were no differences in the uterine microbiome at calving, omics integration was only performed at diagnosis. Statistical significance was considered at *P* ≤ 0.05.

## Results

### Microbiome

**Fig. 1:**
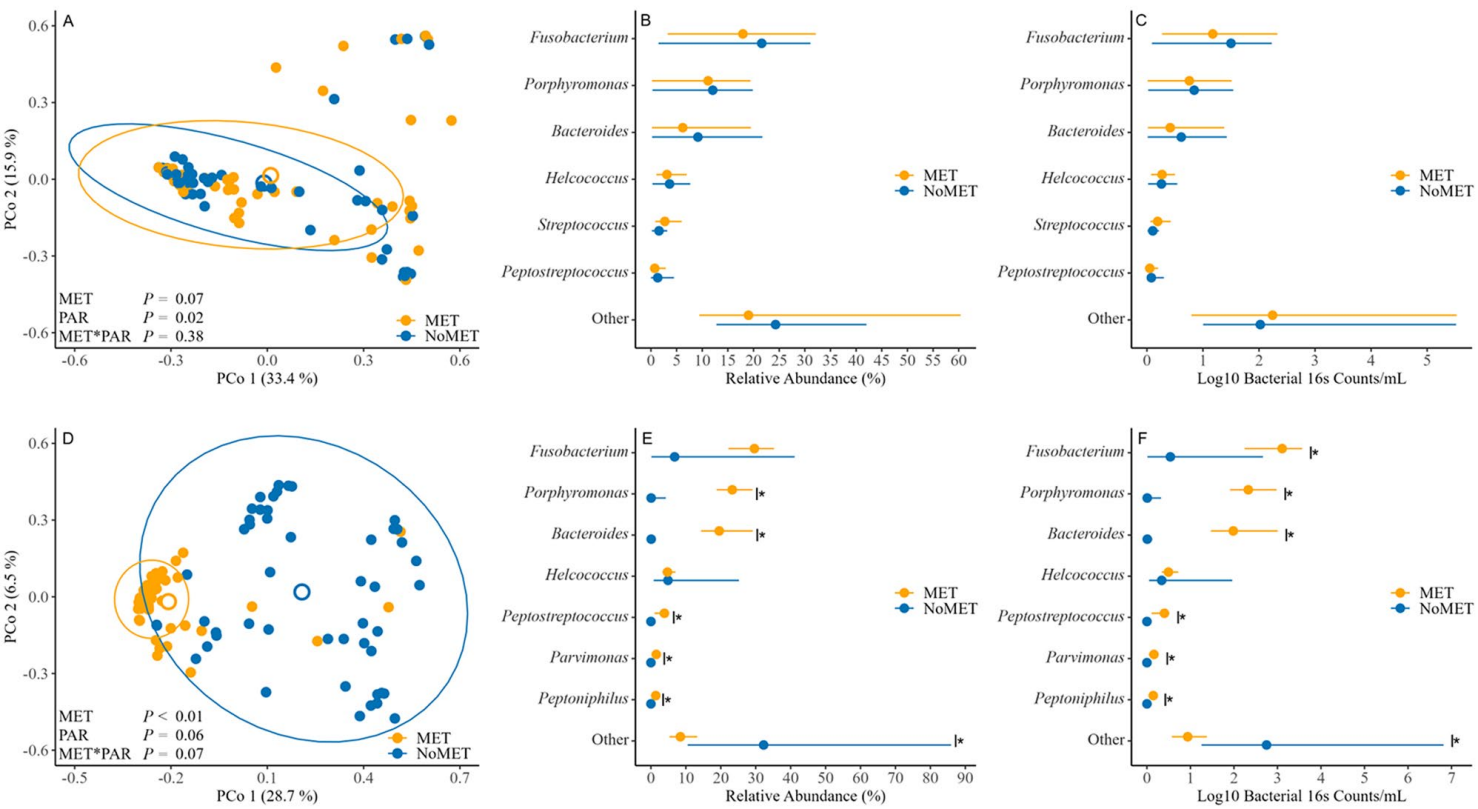
Comparison of uterine microbiome at a genus level at calving (**A**, **B**, **C**) and on the day of metritis diagnosis (3, 7, or 10 days after calving; **D**, **E**, **F**) between dairy cows that developed metritis (MET; orange; *n* = 52) and dairy cows that did not develop metritis (NoMET; blue; *n* = 52). The uterine microbiome was identified by amplification of the V4 hypervariable region of the bacterial/archaeal 16 S rRNA. Panel A and D: results from principal coordinate analyses with Bray-Curtis distances at calving and at metritis diagnosis, respectively. Percentages within each principal component (PCo) correspond to the percentage of variation explained by the component. The ellipses correspond to 95% confidence intervals. *P*-values correspond to permutational analysis of variance (PERMANOVA) based on Bray-Curtis distances with 9,999 permutations including the effect of metritis (MET vs. NoMet), parity (multiparous vs. primiparous) and their interaction. Full models also included the effects of calving related disorders (CRD; calving *P* = 0.89; diagnosis *P* = 0.66), interaction between metritis and CRD (calving *P* = 0.90; diagnosis *P* = 0.97), interaction between parity and CRD (calving *P* = 0.22; diagnosis *P* = 0.33), and interaction between metritis, parity, and CRD (calving *P* = 0.32; diagnosis *P* = 0.53). The model on the day of metritis diagnosis also contained the effect of day of metritis diagnosis (d3, d7, d10; *P* = 0.20); Panel B, C, E, F: individual bacteria genera comparison as relative abundance (**B**, **E**) and as estimated counts (**C**, **F**) at calving and at metritis diagnosis, respectively, between cows that developed metritis and cows that did not develop metritis. Bacteria genera with less than 1% relative abundance were grouped together as “Other”. Significance was tested using Wilcoxon tests with Bonferroni corrections for multiple testing. Effect size was tested using linear discriminant analysis effect size (LEfSe). Circles represent median and lines crossing circles horizontally represent the interquartile range. Asterisks correspond to adjusted *P* < 0.05. Estimated bacterial counts were calculated multiplying the total bacterial 16 S rRNA by the relative abundance of each bacteria genus. Figures were created using the ggplot2 package of Rstudio Version 2023.06.1 + 524 (RStudio, PBC, Boston, MA)

Measures of alpha (Supplemental Figure S1A, B) and beta (Fig. 1A, B and C) diversity at calving did not differ (*P* > 0.05) between cows that did and did not develop metritis.

Cows that developed metritis had greater Shannon (*P* < 0.01; Supplemental Figure S1C) and Simpson (*P* < 0.01; Supplemental Figure S1D) indexes at the day of metritis diagnosis compared with cows that did not develop metritis. The PCoA combined with PERMANOVA showed an association (*P* < 0.01) between metritis and the uterine microbiome structure at the day of metritis diagnosis (Fig. 1D). Cows that developed metritis had greater (*P* < 0.05) relative abundance of *Porphyromonas*, *Bacteroides*, *Parvimonas*, *Peptostreptococcus*, and *Peptoniphilus* than cows that did not develop metritis at the day of metritis diagnosis (Fig. 1E). Cows that developed metritis had greater (*P* < 0.05) estimated absolute counts of *Fusobacterium, Porphyromonas*, *Bacteroides*, *Parvimonas*, *Peptostreptococcus*, and *Peptoniphilus* than cows that did not develop metritis at the day of metritis diagnosis (Fig. 1F). No differences were observed between scores 1 to 4 in total bacteria counts, estimated counts of *Fusobacterium, Porphyromonas*, and *Bacteroides*, and relative abundance of *Fusobacterium, Porphyromonas*, and *Bacteroides*. Score 5 had greater (*P* < 0.05) total bacteria counts, estimated counts of *Fusobacterium, Porphyromonas*, and *Bacteroides*, and relative abundance of *Porphyromonas* than scores 1 to 4. Score 5 had greater (*P* < 0.05) relative abundance of *Fusobacterium* than score 1 but no differences were observed between score 5 and scores 2 to 4. Score 5 had greater (*P* < 0.05) relative abundance of *Bacteroides* than score 1 to 3 but no differences were observed between score 5 and score 4 (Supplemental Figure S2).

### Metabolome

**Fig. 2:**
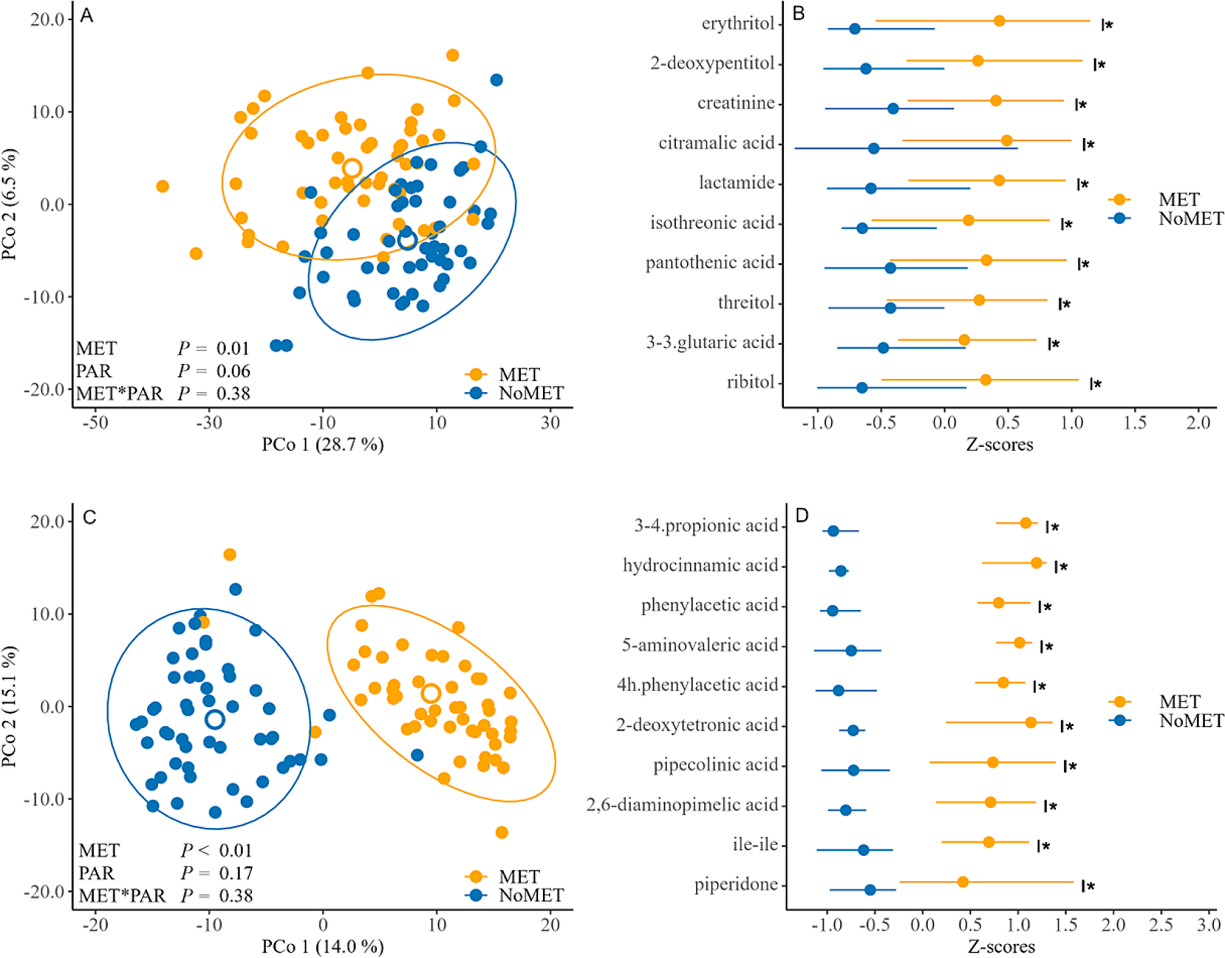
Comparison of uterine metabolome at calving (**A**, **B**) and on the day of metritis diagnosis (3, 7, or 10 days after calving; **C**, **D**) between dairy cows that developed metritis (MET; orange; *n* = 52) and dairy cows that did not develop metritis (NoMET; blue; *n* = 52). The uterine metabolome was identified by gas chromatography time-of-flight mass spectrometry. Panel A and C: results from partial least squares - discriminant analysis (PLS-DA) at calving and at metritis diagnosis, respectively. Percentages within each principal component (PCo) correspond to the percentage of variation explained by the component. The ellipses correspond to 95% confidence intervals. *P*-values correspond to permutational analysis of variance (PERMANOVA) based on Euclidean distances with 9,999 permutations including the effect of metritis (MET vs. NoMet), parity (multiparous vs. primiparous) and their interaction. Full models also included the effects of calving related disorders (CRD; calving *P* = 0.31; diagnosis *P* = 0.73), interaction between metritis and CRD (calving *P* = 0.30; diagnosis *P* = 0.40), interaction between parity and CRD (calving *P* = 0.12; diagnosis *P* = 0.37), and interaction between metritis, parity, and CRD (calving *P* = 0.31; diagnosis *P* = 0.49). The model on the day of metritis diagnosis also contained the effect of day of metritis diagnosis (d3, d7, d10; *P* < 0.01); Panel B and D: top 10 metabolites sorted by effect size resulting from individual metabolite comparisons at calving and at metritis diagnosis, respectively, between cows that developed metritis and cows that did not develop metritis. Significance was tested using Wilcoxon tests with Bonferroni corrections for multiple testing. Effect size was tested using linear discriminant analysis effect size (LEfSe). Circles represent median and lines crossing circles horizontally represent the interquartile range. Asterisks correspond to adjusted *P* < 0.05. 3–3.glutaric acid, 3-hydroxy-3-methylglutaric acid; 3–4.propionic acid, 3-(4-hydroxyphenyl) propionic acid; 4 h.phenylacetic acid, 4-hydroxyphenylacetic acid. Figures were created using the ggplot2 package of Rstudio Version 2023.06.1 + 524 (RStudio, PBC, Boston, MA)

The PLS-DA combined with PERMANOVA showed an association (*P* = 0.01) between metritis and the uterine metabolome structure at calving (Fig. 2A). Evaluation of individual metabolites revealed that 29 metabolites differed (*P* < 0.05) between cows that did and did not develop metritis (Supplemental Table S2). The 10 most important annotated metabolites driving the difference between cows that developed or did not develop metritis were erythritol, 2-deoxypentitol, creatinine, citramalic acid, lactamide, isothreonic acid, pantothenic acid, threitol, 3-hydroxy-3-methylglutaric acid, and ribitol (Fig. 2B).

There was an association (*P* < 0.01) between metritis and the uterine metabolome structure at the day of metritis diagnosis (Fig. 2C). Evaluation of individual metabolites revealed that 152 metabolites differed (*P* < 0.05) between cows that did and did not develop metritis at the day of metritis diagnosis (Supplemental Table S2). The 10 most important annotated metabolites driving the difference were 3-(4-hydroxyphenyl) propionic acid, hydrocinnamic acid, phenylacetic acid, 5-aminovaleric acid, 4-hydroxyphenylacetic acid, 2-deoxytetronic acid, pipecolic acid, 2,6-diaminopimelic acid, ile-ile, and piperidone (Fig. 2D).

### Uterine integration

Because no differences were observed in the uterine microbiome at calving, uterine microbiome-metabolome integration was not performed.

**Fig. 3:**
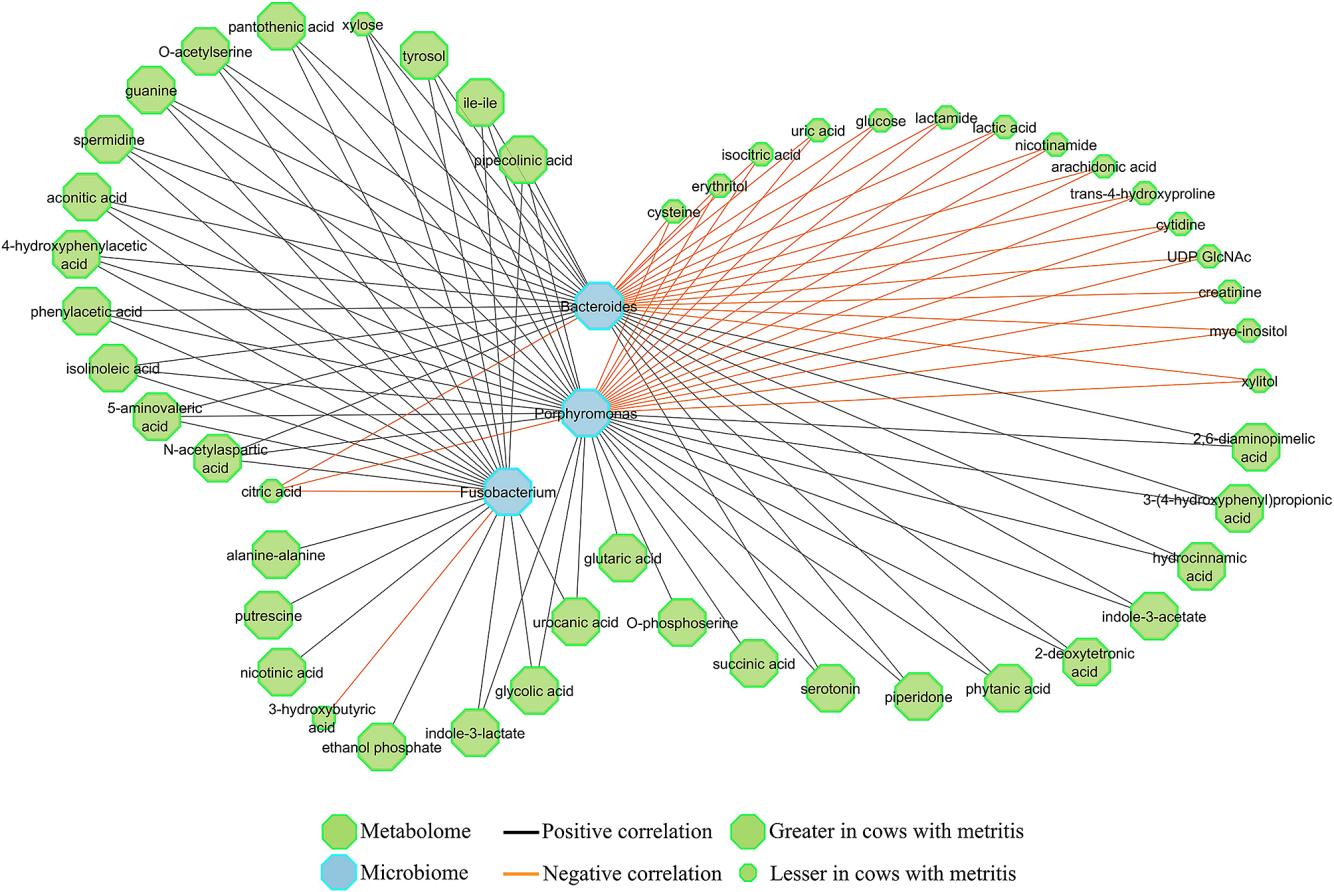
Compound network between bacteria genera and metabolites with the best discriminatory ability between dairy cows that developed metritis (*n* = 52) and dairy cows that did not develop metritis (*n* = 52) on the day of metritis diagnosis (3, 7, or 10 days after calving) according to the sparce partial least squares - discriminant analysis (sPLS-DA). Compound network analysis was performed between bacteria genera and metabolites with a correlation coefficient (*M*) > 0.5 or < -0.5 using the Data Integration Analysis and Biomarker discovery using Latent variable approaches for Omics studies (DIABLO) function of the mixOmics package. Metscape 2 within the CytoScape 3.8 platform was used to edit the figure for easier visualization. Green octagons correspond to metabolites that differed between dairy cows that developed metritis and dairy cows that did not develop metritis, while blue octagons correspond to bacteria genera that differed between dairy cows that developed metritis and dairy cows that did not develop metritis. Large octagons represent metabolites or bacteria genera with greater abundance, while small octagons represent metabolites with lower abundance in the uterine fluid of dairy cows that developed metritis when compared with dairy cows that did not develop metritis. Black lines correspond to positive correlation coefficients while orange lines correspond to negative correlation coefficients

Inclusion of the 6 microbial genera and 152 metabolites identified as statistically different between cows with and without metritis on the day of metritis diagnosis were fed into the sparce PLS-DA model resulting in the selection of 3 genera (*Fusobacterium, Porphyromonas*, and *Bacteroides*) and 120 metabolites as variables for the latent structures. Of the 120 metabolites, 49 had a correlation coefficient (*M*) greater than 0.5 or lesser than − 0.5 with at least one of the 3 microbial genera (Fig. 3). The top 10 metabolites with the greatest median correlation coefficients against the 3 bacteria genera were phenylacetic acid (*M* = 0.70), 4-hydroxyphenylacetic acid (*M* = 0.69), O-acetylserine (*M* = 0.66), pipecolic acid (*M* = 0.66), tyrosol (*M* = 0.65), 3-(4-hydroxyphenyl) propionic acid (*M* = 0.65), hydrocinnamic acid (*M* = 0.65), 5-aminovaleric acid (*M* = 0.64), 2,6-diaminopimelic acid (*M* = 0.64), and 2-deoxytetronic acid (*M* = 0.63). All of these, except for 4-hydroxyphenylacetic acid, O-acetylserine, and tyrosol were also among the top 10 most important annotated metabolites that differed between cows with and without metritis according to Wilcoxon and LEfSe results. Although not among the top 10 metabolites, Arachidonic acid (*M* = -0.61), nicotinamide (*M* = -0.58), and citric acid (*M* = -0.57) had the strongest negative correlation with the 3 bacteria genera. The complete correlation matrix can be found in Supplemental Table S3.

## Discussion

**Fig. 4:**
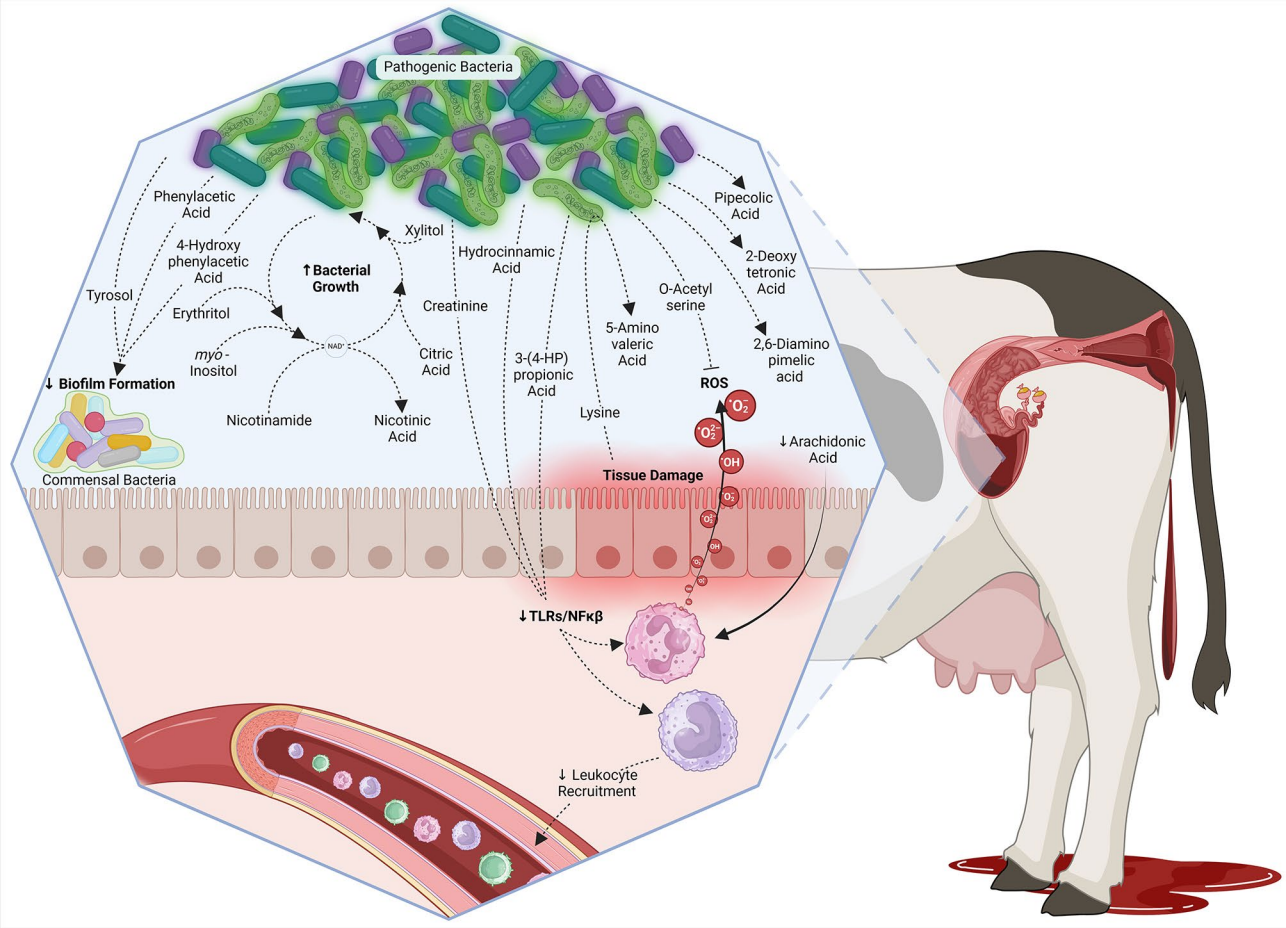
Illustration of the possible role of metabolites in the uterine crosstalk with opportunistic pathogenic bacteria on the day of metritis diagnosis. Altogether, from the 49 metabolites with a strong correlation with opportunistic pathogenic bacteria on the day of metritis diagnosis, the 17 metabolites illustrated herein have been described as part of processes associated with attenuation of biofilm formation by commensal bacteria, opportunistic pathogenic bacterial overgrowth, tissue damage and inflammation, immune evasion, and immune dysregulation. Down arrows indicate reduction, and up arrows indicate increase in cows with metritis. NAD^+^, nicotinamide adenine dinucleotide; HP, hydrophenyl; ROS, reactive oxygen species; TLR, toll-like receptor; NFκβ, nuclear factor kappa beta. Figure created with Biorender

Herein, uterine microbiome and metabolome at calving and at metritis diagnosis were compared between cows that did and did not develop metritis. Then, differential bacteria genera and metabolites were used to assess their intra-omics correlation aiming to advance our understanding of the uterine environment in dairy cows with metritis. Because no differences in the microbiome were found at calving, data integration was not performed at calving. At the day of metritis diagnosis, the top 10 metabolites with the greatest median correlation coefficients against *Fusobacterium, Porphyromonas*, and *Bacteroides* are discussed, all of which were positively correlated with these bacteria. All of these, except for 4-hydroxyphenylacetic acid, O-acetylserine, and tyrosol were also among the top 10 most important annotated metabolites driving the difference in the uterine metabolome between cows with and without metritis. Furthermore, although not within the top 10, the 3 metabolites with the greatest negative correlation coefficients against these bacteria are discussed. These are arachidonic acid, nicotinamide, and citric acid. Lastly, differentially abundant metabolites at calving that were also differentially abundant at metritis diagnosis are discussed. Discussed metabolites can be found in Fig. 4.

Within the 10 most important metabolites correlated with the most important bacteria at metritis diagnosis, phenylacetic acid and 4-hydroxyphenylacetic acid derive from microbial fermentation of aromatic amino acids, a process particularly prevalent among *Bacteroides* [26, 27]. Phenylacetic acid has been described to disrupt quorum sensing and attenuate biofilm formation by *Pseudomona aeruginosa* [28] and inhibit growth of *Staphylococcus aureus* and *Escherichia coli* [29]. Furthermore, *Bacteroides* has been shown to produce phenylacetic acid and 4-hydroxyphenylacetic acid from aromatic amino acids in the absence of glucose. Conversely, bacteria previously associated with a healthy uterus, such as *Firmicutes* [6] need glucose to ferment aromatic amino acids [26]. Herein, glucose was lower in the uterine fluid of cows with metritis; therefore, it is possible that the growth and proliferation ability of *Bacteroides* and other bacteria associated with metritis may be in part mediated by their ability to proliferate in an environment where glucose is lacking. Furthermore, the byproducts of aromatic amino acid metabolism may help opportunistic pathogenic bacteria thrive over uterine commensals.

O-acetylserine is a key intermediate metabolite for de-novo cysteine production by microbes [30], which is used for glutathione production, a major cellular antioxidant [31]. *Salmonella typhimurium* incapable of synthesizing O-acetylserine has been shown to produce less cysteine, which lead to increased susceptibility to oxidative stress [31]. The greater abundance of O-acetylserine in cows with metritis together with the strong positive correlation with opportunistic pathogenic bacteria suggests that these bacteria may produce O-acetylserine as a defense mechanism against the reactive oxygen species produced by immune cells. On the other hand, biofilm formation by *Escherichia coli and Providencia stuartii* was reduced when O-acetylserine was added to the culture media [32], indicating that O-acetylserine may expose bacteria to the immune system by decreasing biofilm formation. It is plausible, therefore, that opportunistic pathogenic bacteria like *Fusobacterium*, *Porphyromonas*, and *Bacteroides* have a greater production of O-acetylserine compared with uterine commensals, which would protect them against reactive oxygen species in an environment with decreased protection from biofilm.

Pipecolic acid is a cyclic amino acid product of lysine degradation by microbes [33]. Bacteria subjected to hyperosmotic stress degrade lysine, leading to an increase in pipecolic acid production, which is believed to contribute to salt tolerance [34]. The uterus of a dairy cow with metritis can be considered a challenging environment given the strong immune response against pathogens [35]. It is not clear why pipecolic acid is increased in cows with metritis but its strong positive correlation with opportunistic pathogenic bacteria suggests that the production of pipecolic acid may be a response mechanism to inflammation in the uterus.

Tyrosol is a phenolic compound derived from microbial fermentation of tyrosine [36] that has been shown to reduce biofilm formation by gram-positive bacteria [37–39] and ATP production by *Escherichia coli* [40]. Independent free-floating bacterial cells, not protected by biofilm, are more susceptible to nutrient deprivation and phagocytosis [41]. *Fusobacterium* is one of the few bacteria able to produce phenols from tyrosine [42]; therefore, it is possible that *Fusobacterium* produces tyrosol to impair the growth of competing bacteria, thereby promoting its own proliferation and dominance in the microbial ecosystem.

Hydrocinnamic acid and its metabolite 3-(4-hydroxyphenyl) propionic acid are two bacterial metabolites typically associated with bacterial fermentation of plant cell wall components [43]. In humans, hydrocinnamic acids are associated with intestinal protective effects by modulating intestinal immunity [44, 45], downregulating the activation of TLR- 4/NF-κβ [46], and by increasing the Bacteroidota/Bacillota ratio [47]. Although in humans hydrocinnamic acids are associated with a healthy intestinal environment, in the context of the bovine uterus, a greater abundance of Bacteroidota is associated with uterine disease [6, 7, 48]. Downregulation of the immune response against uterine pathogens may predispose dairy cows to metritis development. We have previously shown that cows with metritis had a reduction in monocyte and T-helper activation in the peripheral blood [8], and plasma metabolomic changes associated with a reduction in leukocyte activation on the day of metritis diagnosis [9]. We hypothesize that the greater levels of hydrocinnamic acids, potentially produced by opportunistic pathogenic bacteria, may be contributing to the immune dysregulation observed in the peripheral blood of cows with metritis.

5-aminovaleric acid is another byproduct of lysine metabolism [49]. Elevated levels of urinary 5-aminovaleric acid have been associated with intestinal inflammation [50]. Lin et al. (2010) proposed that higher levels of 5-aminovaleric may be caused by inflammation-induced tissue damage, which increases the levels of lysine for intestinal microbiota and the release of 5-aminovaleric acid [50]. Cows with metritis have extensive endometrial damage and inflammation [35] which may provide lysine for bacterial degradation, increasing 5-aminovaleric acid levels in the uterine fluid of cows that developed metritis.

2,6-Diaminopimelic acid is a bacterial cell wall peptidoglycan component found in gram-negative bacteria [51]; therefore, an increase in 2,6-diaminopimelic acid reflects the greater *Fusobacterium, Porphyromonas*, and *Bacteroides* abundance in the uterus of cows with metritis.

2-deoxytetronic acid is a sugar acid derivative that acts as an appetite suppressant in rats [52] and has been associated with subclinical ketosis in dairy cows [53]. The role of 2-deoxytetronic acid in the crosstalk between microbes and metabolites in the uterus of cows that develop metritis is unclear and deserves further investigation.

Although not within the top 10 metabolites with the greatest absolute correlation with opportunistic pathogenic bacteria, it is also important to mention the metabolites with the greatest negative correlation with opportunistic pathogenic bacteria because these could potentially be used to reduce the abundance of these bacteria in the uterus. Arachidonic acid is a polyunsaturated fatty acid commonly known as a rate-limiting precursor to the synthesis of biologically active eicosanoic acids such as prostaglandins, thromboxanes, and leukotrienes [54]. Since the intracellular availability of arachidonic acid is a bottleneck in the biosynthesis of eicosanoic acids [55], immune cells can uptake extracellular arachidonic acid to aid in the production of eicosanoic acids [55, 56]. Therefore, it is plausible that arachidonic acid is being taken up by immune cells to mount an inflammatory response against pathogens.

Nicotinamide, a form of vitamin B3, is used by bacteria to form nicotinamide adenine dinucleotide (NAD^+^), releasing nicotinic acid to the extracellular space [57]. Bacteria use NAD^+^ as a co-factor for several enzymes involved in ATP production and bacterial survival [58]. Interestingly, not only nicotinamide had a strong negative correlation with opportunistic pathogenic bacteria genera, but also nicotinic acid had a strong positive correlation with opportunistic pathogenic bacteria (*M* = 0.50), suggesting that opportunistic pathogenic bacteria may use nicotinamide as a precursor for NAD^+^, boosting microbial growth and survival. Furthermore, given that citric acid is an important substrate for energy production by bacteria [59], the strong negative correlation between citric acid and nicotinamide with opportunistic pathogenic bacteria indicates that these substrates may be used for their growth.

From the 49 metabolites with a strong correlation with *Fusobacterium, Porphyromonas*, and *Bacteroides* on the day of metritis diagnosis only 7 also differed at calving. Interestingly, of those 7 metabolites, erythritol, xylitol, *myo*-inositol, creatinine, and lactamide were greater at calving but lesser at the day of diagnosis. Erythritol, xylitol [60, 61] and *myo*-Inositol [62] can be utilized by bacteria for growth and proliferation [63]; therefore, their greater abundance at calving and lesser abundance at the day of metritis diagnosis in cows that developed metritis indicates that opportunistic pathogenic bacteria may be using these metabolites as substrates. Creatinine is a byproduct of muscle metabolism that when exposed to mouse macrophages, leads to a sharp reduction in TLR-2, -3, -4, and − 7 transcript levels [64]. Toll-like receptors are involved in sensing the presence of bacterial antigens prior to their phagocytosis and killing [64]. It is possible that the greater levels of creatinine at calving in cows that developed metritis may be contributing to a poorer immune response against opportunistic pathogenic bacteria, leading to their overgrowth. Lactamide is an acyl amide derivative from the amidation of lactic acid [65]; however, lactamide metabolism and role in bacterial proliferation is not clear and deserves further investigation.

## Conclusions

This observational study provides insights into the uterine microbiome and metabolome at calving and on the day of metritis diagnosis in cows that developed metritis. Furthermore, this is the first-time uterine crosstalk between opportunistic pathogenic bacteria and metabolites on the day of metritis diagnosis has been explored. Altogether, from the 49 metabolites with a strong correlation with opportunistic pathogenic bacteria on the day of metritis diagnosis, the 17 metabolites discussed herein have been described as part of processes associated with attenuation of biofilm formation by commensal bacteria, opportunistic pathogenic bacteria overgrowth, tissue damage and inflammation, immune evasion, and immune dysregulation. The data integration presented herein helps advance the understanding of the uterine environment in dairy cows with metritis. Furthermore, the main metabolites described herein may be promising targets for future interventions aiming to reduce opportunistic pathogenic bacteria growth in the uterus, and therefore, reducing the incidence of metritis.

### Electronic supplementary material

Below is the link to the electronic supplementary material.

Supplementary Material 1

Supplementary Material 2

Supplementary Material 3

Supplementary Material 4

## Data Availability

The raw sequence data generated during this study are available in the NCBI repository under BioProject OR883023 (www.ncbi.nlm.nih.gov/nuccore/OR883023) - OR883397 (www.ncbi.nlm.nih.gov/nuccore/OR883397).The metabolomics dataset analyzed during the current study is available in the NIH Common Fund’s National Metabolomics Data Repository website, the Metabolomics Workbench repository [66] under Study ID ST002994, 10.21228/M8S425. The Metabolomics Workbench is supported by NIH grant U2C-DK119886 and OT2-OD030544 grants.

## Abbreviations

PCoA: Principal coordinate analysis
PLS-DA: Partial least square – discriminant analysis
TMR: Total mixed ration
BCS: Body condition score
BW: Body weight
VDS: Vaginal discharge score
CRD: Calving related disorders
ASV: Amplicon sequence variant
PERMANOVA: Permutational analysis of variance
LEfSe: Linear discriminant analysis effect size
DIABLO: Data integration analysis and biomarker discovery using latent variable approaches for omics studies
*M*: Correlation coefficient
LDA: Linear discriminant analysis score
NAD^+^: Nicotamide adenine dinucleotide
