# Supplementary material for: Integrating uterine microbiome and metabolome to advance the understanding of the uterine environment in dairy cows with metritis
Source: Anim Microbiome. 2024 May 27;6:30. doi: 10.1186/s42523-024-00314-7 (PMC11131188; doi:10.1186/s42523-024-00314-7)
Supplement: Supplementary file 4 — Supplementary Material 4 [file 42523_2024_314_MOESM4_ESM.docx]

**SUPPLEMENTAL FIGURES**

**Integrating uterine microbiome and metabolome to advance the understanding of the uterine environment in dairy cows with metritis**

S. Casaro^1^, J. G. Prim^2^, T. D. Gonzalez^1^, F. Cunha^1^, R. S. Bisinotto^1^, R. C. Chebel^1^, J. E. P. Santos^3,4^, C. D. Nelson^3^, S. J. Jeon^5^, R. C. Bicalho^6^, J. P. Driver^7^, K. N. Galvão^1,4^*

^1^Department of Large Animal Clinical Sciences, University of Florida, Gainesville, FL

^2^Department of Clinical Sciences, Auburn University, Auburn, AL

^3^Department of Animal Sciences, University of Florida, Gainesville, FL

^4^D. H. Barron Reproductive and Perinatal Biology Research Program, University of Florida, Gainesville, FL

^5^Department of Veterinary Biomedical Sciences, Long Island University, Brookville, NY

^6^FERA Diagnostics and Biologicals, College Station, TX

^7^Division of Animals Sciences, University of Missouri, Columbia, MO

*Corresponding author: Klibs N. Galvão: [galvaok@ufl.edu](mailto:galvaok@ufl.edu)

Declarations of interest: none


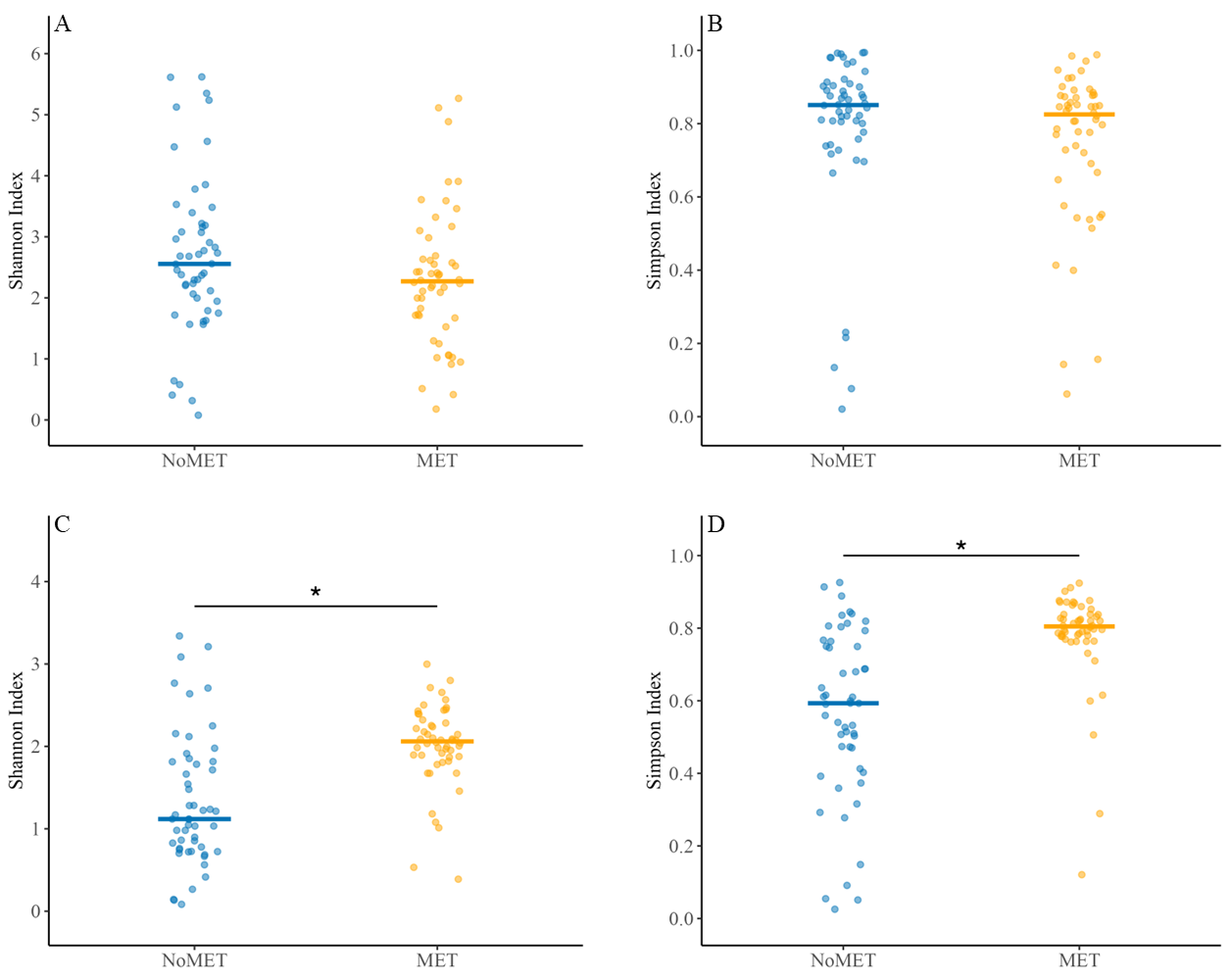
Supplemental Figure S1. Comparison of uterine microbiome alpha diversity at calving (A, B) and on the day of metritis diagnosis (3, 7, or 10 days after calving; C, D) between dairy cows that developed metritis (MET; orange; n = 52) and dairy cows that did not develop metritis (NoMET; blue; n = 52). The uterine microbiome was identified by amplification of the V4 hypervariable region of the bacterial/archaeal 16S rRNA. Horizontal lines represent median value for each group. The effect of metritis was analyzed on each index using the Wilcoxon test. Asterisks correspond to P < 0.05. Figures were created using the ggplot2 package of Rstudio Version 2023.06.1+524 (RStudio, PBC, Boston, MA).


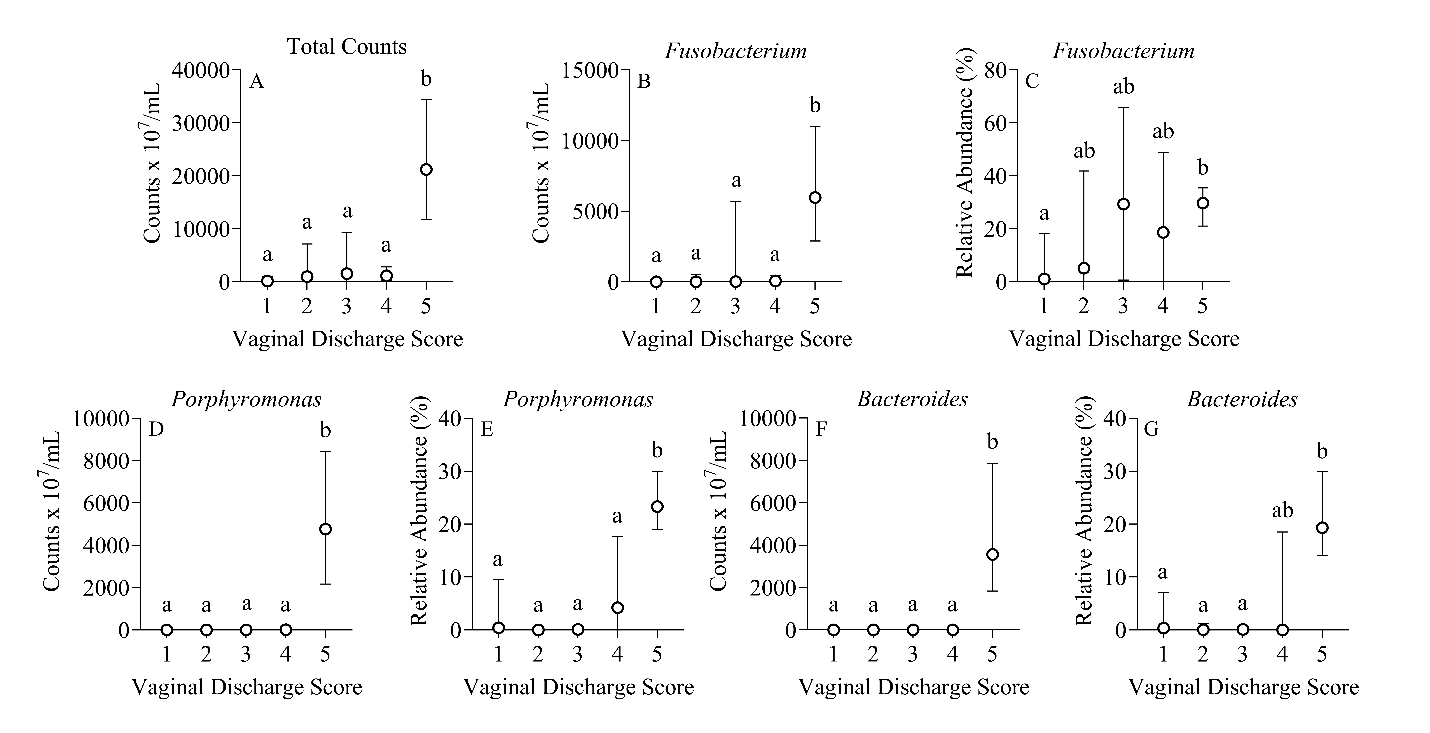
 Supplemental Figure S2. Comparison of the three most important uterine bacteria at a genus level on the day of metritis diagnosis (3, 7, or 10 days after calving) between dairy cows with different vaginal discharge scores. Vaginal discharges were collected with a Metricheck device (Metricheck, Simcro, New Zealand) and categorized as 1 (not fetid normal lochia, viscous, clear, red, or brown; n = 14), 2 (cloudy, pink, red, or brown mucoid discharge with flecks of pus; n = 18), 3 (not fetid, pink red or brown mucopurulent discharge with < 50% pus; n = 13), 4 (not fetid, pink, red or brown purulent discharge with ≥ 50% pus; n = 7), and 5 (fetid red-brownish, watery discharge; n = 51). The uterine microbiome was identified by amplification of the V4 hypervariable region of the bacterial/archaeal 16S rRNA. Estimated bacterial counts were calculated multiplying the total bacterial 16S rRNA by the relative abundance of each bacteria genus. Total counts, individual bacteria genera estimated counts, and individual bacteria genera relative abundance were compared between the different vaginal discharge scores using Bonferroni corrected Wilcoxon tests. Circles represent median and lines crossing circles vertically represent the interquartile range. Different letters correspond to adjusted P < 0.05 between groups. Figures were created using GraphPad Prism Version 8.4.3 (686).
